# Hemoperfusion with CytoSorb^®^ in Pediatric Patients: A Monocentric Case Series

**DOI:** 10.3390/jcm13216587

**Published:** 2024-11-01

**Authors:** Eva Rihar, Vanja Peršič, Alexander Jerman, Tina Plankar Srovin, Gorazd Mlakar, Neva Bezeljak, Marko Pokorn, Petja Fister

**Affiliations:** 1 University Children’s Hospital, University Medical Center Ljubljana, 1000 Ljubljana, Slovenia; 2 Center for Acute and Complicated Dialysis and Vascular Access, Department of Nephrology, University Medical Center Ljubljana, 1000 Ljubljana, Slovenia; 3 Faculty of Medicine, University of Ljubljana, 1000 Ljubljana, Slovenia; 4 Department of Infectious Diseases, University Medical Center Ljubljana, 1000 Ljubljana, Slovenia; 5 Unit of Cardiology, University Children’s Hospital, University Medical Center Ljubljana, 1000 Ljubljana, Slovenia; 6 Department of Pediatric Intensive Care, University Children’s Hospital, University Medical Center Ljubljana, 1000 Ljubljana, Slovenia

**Keywords:** CytoSorb, hemoperfusion, hemoadsorption, child, neonate, sepsis, cytokine storm

## Abstract

**Background:** Pro- and anti-inflammatory cytokines help regulate the inflammatory response. A cytokine storm is a dysregulated cytokine response associated with sepsis and other conditions that result in a hyper-inflammatory state. Extracorporeal cytokine adsorption has the potential to restore a balanced inflammatory response. Hemoperfusion with CytoSorb^®^ (CS) appears to be a short-term mode of treatment of sepsis in both adults and children. **Objective:** We present a case series of eight critically ill children involving the use of hemoperfusion with CS at the University Medical Center Ljubljana to treat clinically manifested cytokine storm. **Results:** In our preliminary experience, it was applied without complication in five children weighing more than 10 kg, who all survived. The effect of age on complications regarding CS treatment is unclear, yet in our case series, all three patients weighing less than 10 kg died of their disease. **Conclusions:** In our preliminary experience, cytokine adsorption with CS remains a potential adjunctive treatment for cytokine storm in critically ill children.

## 1. Introduction

Cytokines are small, soluble proteins that have regulating effects on the immune response. A balanced recruitment of pro-inflammatory (interleukin (IL)-1β, IL-6, IL-8, IL-12, and tumor necrosis factor (TNF) α) and anti-inflammatory cytokines (IL-4, IL-10, IL-13, and IL-1 receptor antagonist (IL-1RA)) maintain immune homeostasis [1,2]. Immunological disturbances occur in states of severe systemic inflammation, leading to an excessive or uncontrolled immune response [3] known as cytokine storm [1]. The potential for cytokine storm and a life-threatening hyper-inflammatory condition is associated with sepsis, severe inflammatory response from viral infections, autoimmune diseases, graft-versus-host disease (GVHD), acute pancreatitis, major trauma, and major surgery [4]. Cytokine storm results in cytokine-induced cell dysfunction and compensatory anti-inflammatory response syndrome (CARS, also called immunoparalysis) [5] that can continue even after the cytokine storm abates [6].

CytoSorb^®^ (CS) is an adsorption cartridge used for removing excess cytokines and other solutes (e.g., myoglobin, bilirubin) from the bloodstream [1,7,8]. Solute removal is based on size exclusion and surface adsorption [9]. The goal of CS application is to reduce cytokines to levels that are no longer harmful while keeping the immune system intact [1]. CS targets hydrophobic molecules of 5–60 kDa (leaving out bacterial endotoxins [2]), while solutes outside this size range (electrolytes and immune cells) supposedly remain unchanged. The efficacy of CS is concentration-dependent, which means that cytokines in higher concentrations are eliminated faster [1]. This appears to be important because mortality is highest when pro- and anti-inflammatory cytokine levels are highest [10]. Attempts to target individual immunomodulatory components might have been unsuccessful [3] due to the redundancy of the cytokine system with several cytokines having similar functions [1]. As hemoadsorption evolved toward the non-specific removal of inflammatory mediators, outcomes became more encouraging [3].

CS can be applied as stand-alone therapy or in combination with other extracorporeal circuits [1]. The typical duration of CS therapy is two to seven consecutive days [11]. A single CS cartridge can be used for up to 24 h in adults, before replacement if the treatment indication persists [1]. More frequent replacement would be sensible when suspecting CS saturation, indicated by the absent reduction in IL-6 concentration pre- and post-CS cartridge [12], or by a rebound increase in the vasopressor dose after eight hours of CS, followed by tapering down after changing the CS cartridge [13]. Concomitant removal of the relevant drug therapy (certain antimicrobials, anticoagulants, sedatives) should be considered as well, as it allows the clinician to increase the initial dose and/or giving a supplementary dose one to two hours after initial CS therapy [14].

In adults, the major indications for CS are septic shock and systemic inflammatory response syndrome (SIRS) after cardiopulmonary bypass (CPB) [1]. A few adult studies of septic shock treated with a combination of CS and renal replacement therapy (RRT) have shown hemodynamic improvement, with or without a reduction in serum concentrations of inflammatory mediators [12,15,16,17]. Few case reports of CS therapy in children have also been published, and no large randomized clinical trial (RCT) examining CS in children has been carried out so far. In children and adults, CS has been predominantly used as an adjunctive therapy to RRT in septic shock management. The reports in children appear promising and show hemodynamic improvement, a decreased concentration of inflammatory markers, and good survival [18,19,20,21,22,23,24,25]. This appears important as sepsis and septic shock remain major global health burdens with high rates of morbidity and mortality in adults [26] and children [27]. At University Medical Center Ljubljana, cytokine adsorption therapy with CS has been used for critically ill children with cytokine storm since 2018. The aim of our case series is to present our pediatric experience with CS.

## 2. Materials and Methods

### 2.1. Patients and Study Design

We performed a retrospective analysis of all children treated with CS in the pediatric intensive care unit (PICU) in University Medical Center Ljubljana, Slovenia, from September 2018 to February 2020. The study complied with the Declaration of Helsinki (as revised in Fortaleza 2013) and was approved by the National Medical Ethics Committee (No. 0120-533/2019/5). Consent was waived due to the retrospective nature of the study. We gathered epidemiological data (age, sex, body mass, and indication), intensive treatment parameters (mechanical ventilation, vasopressor use, inotrope use, acute kidney injury (AKI), extracorporeal membrane oxygenation (ECMO)), procedure-specific parameters (blood access, duration, blood and dialysate flow rates, dialysis dose, adverse events, device malfunction), and treatment outcome. The primary outcome measures in our report were the effects of CS on inflammatory markers (C-reactive protein (CRP), procalcitonin (PCT), IL-6) and clinical status (vasopressor-inotrope requirement, survival rates). The efficacy of the treatment was evaluated every three to six hours through close monitoring of these parameters. CS was discontinued once hemodynamic stabilization was achieved and when inflammatory markers showed at least a trend of improvement, or in cases where the patient’s clinical condition deteriorated.

### 2.2. CytoSorb^®^ Treatment

There is no standardized protocol for initiation and duration of treatment with CS at our institution. However, based on positive outcomes with CS in adults, we decided to extend its use to pediatric patients. Upon agreement between an intensive care pediatrician, a nephrologist, and a pediatric infectious diseases specialist, we initiated CS in carefully selected patients suspected of having on-going cytokine storm. The procedure was started (a) within 12–24 h in patients with suspected fulminant meningococcemia and necrotizing fasciitis, and (b) in patients who showed deterioration after a “stabilization period” intended to assess the effect of standard therapy, including those with refractory septic shock, GVHD with SIRS, and acute liver failure (ALF) as a part of gestational alloimmune liver disease (GALD). Contraindications for CS use were advanced, irreversible medical conditions with a limited life expectancy.

We combined CS and continuous renal replacement therapy (CRRT) in all patients. CS cartridges were placed in the pre-filter position. CRRT was performed with Prismaflex (Gambro, Lund, Sweden) dialysis monitors, a standard pediatric dialysis cartridge (ST60, Prismaflex set), and using automated regional citrate anticoagulation. Blood flow was set to 30–100 mL/min and dialysate flow to 500–1000 mL/h. The dialysis doses were higher than typically recommended as the primary goal was to reduce the cytokine load; the efficiency of cytokine removal by CS is determined by their concentration and the blood flow rate. Therefore, we set the blood flow rate as high as possible and adjusted the citrate dosing accordingly. The desired treatment time was 24 h but was tailored as needed. In cases of a favorable clinical scenario, the treatment was prolonged and the CS cartridge was used for longer than 24 h. Throughout the procedures, careful monitoring of clinical and laboratory parameters was employed to ensure safety and prevent overt disequilibrium due to high dialysis doses.

### 2.3. Data Collection

Baseline patient demographics, intensive care treatment and procedure-related parameters were collected from medical records. Laboratory values were obtained from electronic health records. IL-6 was measured by an electrochemiluminescence assay (Cobas e 411, Roche Diagnostics GmbH, Mannheim, Germany). Vasoactive-inotropic score (VIS) was calculated as follows: dopamine dose (µg/kg/min) + dobutamine dose (µg/kg/min) + 100× norepinephrine dose (µg/kg/min) + 100× epinephrine dose (µg/kg/min) + 10× milrinone dose (µg/kg/min) + 10,000× vasopressin dose (U/kg/min) [28]. The predictive mortality score was calculated for each patient: the score for neonatal acute physiology and perinatal extension II (SNAPPE-II) for newborns [29] and the pediatric index of mortality (PIM-3) for children over one month of age [30].

### 2.4. Statistical Analysis

We performed basic descriptive analysis of the laboratory findings. Due to the small number of measurements, we compared the distributions with the Wilcoxon rank sum test. Since this was a small-number retrospective study, a power analysis was not performed.

## 3. Results

### 3.1. Epidemiological Parameters

We applied CS to eight critically ill children over a period of two years. Their age, gender, body mass, underlying diseases, and indication for CS are reported in Table A1. The median age was 19.5 months (range 0 to 75 months). Half of the children were female. The median body mass was 15 kg (range 1.9 to 27 kg). Predominant indication for CS was septic shock (six of eight patients, i.e., 75%), including meningococcemia in patient No. 3.

### 3.2. Hemodynamic and Respiratory Condition

Patients’ hemodynamic and respiratory condition, necessary cardiorespiratory support during CS therapy, time interval between PICU admission and CS initiation, predictive mortality score on admission to PICU, and actual survival are listed in Table A2. All patients required mechanical ventilation and multiple vasopressors and inotropes; the median VIS was 63 (range 8 to 126) prior to CS treatment and 102 (range 45 to 132) after six hours of CS treatment. Due to cardiocirculatory instability, three patients (38%) required ECMO (all three survived). The median PIM-3 score was 16.9% (range 5.9 to 51.6%), while SNAPPE-II calculated for the newborn patient with GALD was 49, correlating with up to 15% mortality risk. Survival occurred in five of eight children (62%).

### 3.3. Procedure-Related Parameters

Procedure-related parameters are included in Table A3. All patients had central access either by a central venous catheter or ECMO cannulation. The median length of the first CS procedure was 25 h (range 6 to 68 h). The median blood flow during the procedure was 50 mL/min (range 30 to 100 mL/min). The dialysate flow was set to either 500 or 1000 mL/h, the median total effluent reaching 108.33 mL/kg/h (range 62.50 to 500 mL/kg/h).

### 3.4. Laboratory Findings

The serum concentrations of CRP, PCT, IL-6, lactate, pH, hemoglobin, leukocytes, and thrombocytes prior to and six hours after CS initiation are listed in Table A4.

## 4. Discussion

Our retrospective study has shown that CS could be lifesaving in critically ill pediatric patients. We present a case series of eight critically ill children with clinically manifested cytokine storm secondary to septic shock (six patients), GVHD (one patient), or multiorgan failure (MOF) as a part of GALD (one patient). Inflammation control and survival were achieved in five patients (62%).

Pro-inflammatory markers were elevated in all patients before and after CS treatment. Our results did not find a substantial reduction in CRP and PCT concentrations as frequently reported in the literature [15,16,20,21,24,25,31]. This is probably because of a short (six hour) interval between t_1_ and t_2_. Subsequent follow-up of laboratory values might provide accordant results. Some pediatric case reports, however, also found no significant reduction in CRP and PCT after CS [22,32] but reported a significant cytokine removal after 24 h of CS therapy [32]. This is similar to our findings, as IL-6 concentration was elevated in all patients prior to CS and was more than halved after six hours of CS in three patients who all survived. This substantial IL-6 reduction is in accordance with several case reports and retrospective observational studies in children and adults [15,16,19,21,32]. Mehta et al. [15] and Karakitsos et al. [16] found a significant IL-6 reduction in survivors but not in nonsurvivors. In our case series, IL-6 was highest in the nonsurviving infant with acute myeloid leukemia in whom a 10% increase of IL-6 after six hours of CS rescue therapy (started 86 h after PICU admission) was observed. This could be explained by Honoré’s theory that cytokine blood levels remain stable despite effective clearance as interstitial cytokines shift to the bloodstream to restore equilibrium [13]; it is also plausible that in the advanced stage of a full-blown cytokine storm the intervention with CS has limited effect on the observed cytokine reduction. Anyhow, the only large RCT regarding CS treatment found a substantial decrease in IL-6 after a CS treatment of six hours per day for up to seven consecutive days in adult patients with septic shock and ARDS, but the decrease did not differ compared to controls; on the other hand, CS treatment was initiated within 72 h of established sepsis or septic shock and acute lung injury or ARDS [9]. The timing of CS initiation could be crucial for its efficacy—Milella et al. observed improved survival in children treated with CS within 24 h after diagnosis of sepsis [23], similar to some of the studies in septic adults [15,17,33]. In our set of patients, CS was initiated within 24 h of PICU admission in patients No. 3 and 5 who both survived. In all three nonsurvivors, CS was initiated more than 24 h after cytokine storm was determined and it was used as rescue therapy. Randomized controlled studies with different treatment regimens and comparable follow-up of laboratory and hemodynamic variables would perhaps provide a clearer guidance for an optimal CS therapy regime.

According to the principles of pediatric dialysis, the blood volume in the extracorporeal circuit should be less than 10% of the patient’s circulatory volume [34]. A CS dose of 150 mL therefore represents an important part of the newborn’s or infant’s circulatory volume, even more so when RRT or ECMO deduct additional blood volume. In these small children the initiation of CS poses the risks of hemodynamic instability and hemodilution. Significant hypotension can lead to the thrombosis and failure of the venous access, the dialysis machine, or the adsorbent, resulting in additional blood loss. Similar to some of the other reported cases of CS use in pediatric patients [21,22,24,35], we used lower blood flow rates (30–100 mL/min) than in adults (200–350 mL/min). This led us to speculate that the capsule might have lasted longer without reaching saturation. As a result, we extended the duration of the procedure with CS without replacing the capsule for more than 24 h. Using lower blood flow rates, however, potentially increases the risks of thrombosis and technical problems. Even so, in seven patients (87%), no device-related hemodynamic instability, thrombosis, or technical problems occurred. Only the premature newborn with GALD, weighing 1.9 kg, hemodynamically deteriorated shortly after initiation of treatment with CS and CRRT, resulting in resuscitation and preliminary termination of the procedure six hours after onset. The benefit of CS application in patients with low body mass (<10 kg) remains unclear. The lowest body mass reported in children submitted to CS is 3.5 kg and 4 kg in isolated cases, of which the latter survived and the former did not [23,36]. We treated two newborns and one infant weighing 1.9, 2.5, and 8.5 kg, respectively. All three had an unfavorable outcome. The five patients that survived were older than one year and heavier than 10 kg. Most of the literature covering CS treatment in a pediatric population encompasses isolated cases of children weighing 10 kg or more [18,19,20,21,22,23,25,31,32,37,38,39]. However, cases of successful CS treatment were reported in children weighing 5–9 kg with no hemodynamic deterioration [21,23,24,35], although with a transient increase in vasoactive drugs during CS in one case of an infant weighing 9 kg with sepsis and MOF after cardiac surgery [35]. Transient hemodynamic deterioration following device insertion was also observed in two patients weighing 3.5 kg [23] and 14 kg [21]. In our patients with body masses below 10 kg, VIS prior to CS treatment appeared to be low but increased six hours after CS therapy, perhaps as a relatively large portion of their circulatory volume was deducted by the extracorporeal circuits, thereby also diluting vasopressors and inotropes. In children weighing more than 10 kg, VIS prior to CS was high and decreased in two out of five patients six hours after CS treatment initiation. We conclude that the time interval to assess the hemodynamic improvement was too short to observe a substantial decrease in vasoactive and inotropic support needed.

Our group of patients was diverse not only regarding their age and body mass but also their general health condition. Three of them were previously healthy and all of them survived. The other five patients were immunocompromised; two of them were premature, one had previous stem cell transplantation and the remaining two had leukemia. Long-term survival was achieved in four out of five surviving patients. The fifth was a patient who died within a month after CS therapy due to underlying GVHD. The underlying immunodeficiency possibly contributed to the outcome in nonsurvivors.

The adverse effects linked to CS in adults are thrombocytopenia and leukopenia (mild and transient) and clearance of certain antimicrobial therapies [11,14]. To the best of our knowledge, none of the studies describing CS application in children examined its effect on antimicrobial therapy, and only a few of them report its effect on blood count. The findings are not uniform: some report both thrombocytopenia and leukopenia [22,31], some found only thrombocytopenia with a normal leukocyte count [20,39], and still others described no additional reduction in either thrombocyte or leukocyte count [25,35]. In our case series, two patients had lower thrombocyte count after CS: in patient No. 2 the thrombocyte count was halved and could also be attributed to the underlying acute lymphoblastic leukemia or current/recent treatment (e.g., antimicrobial therapy), while in patient No. 8, the thrombocyte count was only a quarter of the count prior to CS and could be a consequence of the on-going fulminant GVHD, ECMO (device-related or heparin-related), antimicrobial therapy, or CS. The three patients with leukopenia prior to CS had a slightly higher concentration after CS, although still in the leukopenic range. The hemoglobin concentration did not markedly change after RRT and CS.

To the best of our knowledge, there is no standardized protocol for CS application in children in terms of CS delay from disease onset, duration of treatment, volumes used, and laboratory parameters to follow. Nevertheless, it appears to be reasonable to start CS therapy early in the course of septic shock to eliminate the excess of cytokines. However, since drug kinetics often change during sepsis and many drugs are removed by CS or RRT, a brief delay in starting CS treatment after administering the initial doses of antibiotics may be necessary to ensure the effectiveness of antibiotic therapy. Although Rugg et al. observed absent benefits of CS treatment in septic shock patients with high serum lactate concentrations [40], our preliminary findings suggest that CS therapy is suitable even in patients with high serum lactate concentrations as many of them survived.

In general, it would perhaps be sensible to base the decision for CS initiation on the ineffectiveness of standard treatment of cytokine storm and the assumption that early CS therapy results in improved survival. It could be worthwhile considering both pro- and anti-inflammatory cytokine dysregulation, as one cannot thoroughly deduce the patient’s response to inflammation or the efficacy of CS treatment from measuring only pro-inflammatory cytokines (e.g., IL-6). Some studies reported CS therapy resulting in significant reduction in other anti-inflammatory cytokines (e.g., IL-10 [31,32], IL-8 [41]) without significantly changing IL-6 concentration. However, as many studies examined the effect of CS on the inflammatory response by measuring IL-6 and found a significant decrease after CS therapy [12,16,19,21,23,32,37], it appears that IL-6 could be an adequate parameter to follow up (but not the only parameter for CS initiation), perhaps as a typical inflammatory response in the acute phase consisting of hyperinflammation [6].

The limitations of our case series include the small number of patients (eight) with diverse indications for CS therapy, the retrospective collection of data, the inability to measure interstitial IL-6 concentrations, and the lack of other cytokine concentration measurements. We were unable to demonstrate the effect of CS application in children with cytokine storm. Finally, the lack of a control group and the complexity of adjunctive patients’ treatment (e.g., steroids, RRT, ECMO) posed difficulties in determining the effect of CS alone on patient outcomes. For instance, we use RRT filters (Prismaflex sets ST60, Gambro, Lund, Sweden) made of AN-69ST material which can bind cytokines [42], meaning that the reduction in IL-6 may be partially attributed to the CRRT procedure itself. On the other hand, ECMO membranes are known to activate the complement cascade, which can trigger cytokine release [43]. As a result, using ECMO in combination with CS could potentially obscure the specific effect of CS.

## 5. Conclusions

In our preliminary experience, cytokine adsorption with CS remains a potential adjunctive treatment for cytokine storm in critically ill children. It was applied without complication in five children weighing over 10 kg, who all survived. The effect of age on complications is unclear. Three patients weighing less than 10 kg died of their disease; immunodeficiency itself might have had an impact on their outcome. Future work measuring interstitial IL-6 or other cytokines is needed to determine the effect of hemoadsorption on cytokine storm.

## Data Availability

The datasets used and/or analyzed during the current study are available from the corresponding author upon reasonable request.

## Appendix A

**Table A1:** Epidemiological parameters.

Patient	1	2	3	4	5	6	7	8
Age (months)	16	66	40	1	23	10	0	75
Gender	M	F	M	F	M	F	F	M
Body mass (kg)	15	20	17	2.5	15	8.5	1.9	27
Underlying disease	WAS, ASCT *	ALL	previously healthy	premature (32 wg), multiple congenital defects ^†^	previously healthy	AML	GALD	previously healthy
Indication for CS^®^	GVHD	septic shock (*Escherichia coli*)	septic shock (*Neisseria meningitidis*), NF	NEC, septic shock (*Escherichia coli*, *Enterococcus faecalis*)	septic shock (*Streptococcus pyogenes*)	septic shock (*Pseudomonas aeruginosa*)	ALF	septic shock (*influenza A virus*)

F, female; M, male; CS, CytoSorb^®^; WAS, Wiskott–Aldrich syndrome; ASCT, allogenic stem cell transplantation; ALL, acute lymphoblastic leukemia; wg, weeks of gestation; AML, acute myeloid leukemia; GALD, gestational alloimune liver disease; GVHD, graft-versus-host disease; NF, necrotizing fasciitis; NEC, necrotizing enterocolitis; ALF, acute liver failure. * 9 months prior to CS treatment; ^†^ operated 1 month prior to CS treatment (tracheoesophageal fistula, esophageal, duodenal and anal atresia, microgastria, persistent cloaca, vertebral anomalies).

**Table A2:** Condition of the patients and the provided cardiorespiratory support.

Patient	1	2	3	4	5	6	7	8
PIM-3 (%)/SNAPPE-II	39.1%	12.8%	5.9%	N/A	51.6%	8.8%	49	21%
Septic shock	no	yes	yes	yes	yes	yes	no	yes
AKI	oliguric	oliguric	no	oliguric	no	oliguric	anuric	oliguric
Mechanical ventilation	yes	yes	yes	yes	yes	yes	yes	yes
Vasoactive medications	A, DA, NA, VP	A, DA, NA, VP	A, DA, DB, NA	A, DA, NA	A, DA, DB, NA	DA, DB, NA, VP	A, DA, VP	A, DA, DB, M, NA
VIS score t_0_	85	98	126	N/A	38	19	8	63
VIS score t_1_	65	102	132	N/A	80	109	115	45
ECMO	no	no	VA	no	VA	no	no	VA
CS^®^ delay (h)	29	106	14	90	9.5	86	28	40.5
Survived	yes	yes	yes	no	yes	no	no	yes

PIM-3, pediatric index of mortality; SNAPPE-II, score for neonatal acute physiology with perinatal extension; AKI, acute kidney injury; A, adrenaline; DA, dopamine; DB, dobutamine; M, milrinone; NA, noradrenaline; VP, vasopressin; ECMO, extracorporeal membrane oxygenation; VA, venoarterial; CS^®^ delay, hours between cytokine storm diagnosis and CytoSorb^®^ initiation; VIS, vasoactive-inotropic score; t_0_, time before initiation of CytoSorb^®^ treatment; t_1_, after six hours of CytoSorb^®^ treatment; N/A, data not available.

**Table A3:** Procedure-related parameters.

Patient	1	2	3	4	5	6	7	8
Vascular access	CVC	CVC	ECMO	CVC	ECMO	CVC	CVC	ECMO
1st procedure duration (h)	47	31	68	19	N/A	18	6	N/A
Blood flow (mL/min)	75	50	50	30	N/A	50	30	100
Dialysate flow (mL/h)	500	500	500	500	N/A	500	500	1000
Total effluent (dialysis dose) (dialysate + citrate + ultrafiltration volume) (mL/kg/h)	108.33	62.50	73.53	380.00	N/A	147.06	500.00	92.59

CVC, central venous catheter; ECMO, extracorporeal membrane oxygenation; N/A, data not available.

**Table A4:** Laboratory findings before and after CytoSorb^®^ treatment.

Patient	1	2	3	4	5	6	7	8	Median	*p* Value ^1^
CRP t_0_ (mg/dL)	182	182	38	159	112	311	26	26	136	0.7
CRP t_1_ (mg/dL)	180	171	53	47	178	153	6	54	104	
PCT t_0_ (ng/mL)	21.7	9.7	70.4	13.1	89.1	73.3	1.3	N/A	22	>0.9
PCT t_1_ (ng/mL)	18.3	8.3	54.5	19.7	959	5.9	N/A	26.4	20	
IL-6 t_0_ (pg/mL)	>5000	5900	>5000	884	>5000	31,120	772	318	5000	0.9
IL-6 t_1_ (pg/mL)	2529	2474	1908	N/A	>5000	34,389	N/A	N/A	2529	
Lactate t_0_ (mmol/L)	12.6	11.9	9.9	2.1	3.9	5.5	3.6	10.5	7.7	0.8
Lactate t_1_ (mmol/L)	12.1	9.5	11.2	5.7	2.5	10.5	9.5	5.3	9.50	
pH t_0_	7.3	7.34	7.17	7.07	7.47	7.39	7.13	7.32	7.31	0.5
pH t_1_	7.31	7.31	7.15	7.19	7.35	7.07	6.8	7.35	7.25	
Hemoglobin t_0_ (g/L)	101	113	158	103	104	125	108	105	106	0.3
Hemoglobin t_1_ (g/L)	111	118	154	134	123	115	81	108	116	
Leukocytes t_0_ (10^9^/L)	8	0.2	16.7	11.6	1.4	0.5	20.3	11.3	10	0.6
Leukocytes t_1_ (10^9^/L)	14.1	0.3	16.9	22.6	4.8	3.2	17.3	7.6	11	
Thrombocytes t_0_ (10^9^/L)	36	50	64	14	107	11	67	118	57	0.8
Thrombocytes t_1_ (10^9^/L)	49	26	115	24	116	20	63	30	40	

t_0_, the time shortly before the initiation of CytoSorb^®^ treatment; t_1_, after six hours of CytoSorb^®^ treatment; N/A, data not available. ^1^ Wilcoxon rank sum test.
